# Early Life Exposure to Violence: Developmental Consequences on Brain and Behavior

**DOI:** 10.3389/fnbeh.2019.00156

**Published:** 2019-07-09

**Authors:** Isabelle Mueller, Ed Tronick

**Affiliations:** ^1^Developmental and Brain Sciences Program, Department of Psychology, University of Massachusetts Boston, Boston, MA, United States; ^2^Department of Newborn Medicine, Harvard Medical School, Boston, MA, United States

**Keywords:** intimate partner violence, domestic violence, infancy, development, brain development

## Abstract

Exposure to intimate partner violence (IPV) can have long-lasting effects on a child’s socio-emotional and neurological development. Research has focused on the effects of IPV on women or older children, while the developmental consequences of exposure to domestic violence during early childhood are less well documented. However, one would expect significant developmental effects since the infant’s brain and stress-related systems are especially susceptible to environmental stimuli. The goal of this mini-review is to examine how findings on infant exposure to IPV can be related to risk and resilience of development in infancy. We describe the known effects of witnessing violence during the perinatal period on socio-emotional development and the possible pathways by which IPV affects brain and stress-regulating systems. Exposure to IPV during infancy disrupts the infant’s emotional and cognitive development, the development of the Hypothalamus-Pituitary-Adrenal (HPA) axis and brain structures related to witnessing itself (auditory and visual cortex). The findings are embedded in the context of the resource depletion hypothesis. A central problem is the dearth of research on exposure to IPV during infancy, its effect on caregiving, and infant development. Nonetheless, the available evidence makes it clear that policies for prevention of IPV are critically needed.

## Introduction

The long-term consequences of exposure to adversity in infancy have been well documented, especially its effects on a child’s development and increased vulnerability for later mental health problems (Kessler et al., 2010; Carr et al., 2013; Reuben et al., 2016). While adversities such as chronic neglect or abuse have been extensively described in the literature, the negative consequences of exposure to intimate partner violence (IPV) are less well documented. Primary reasons for the dearth of findings are that cases of IPV often go unreported out of fear of consequences or get palliated by the individuals involved. IPV as defined by the Centers for Disease Control and Prevention is one intimate partner exercising coercive control over the other, including physical and sexual violence, as well as threats of physical or sexual violence, and emotional abuse in the context of physical and sexual violence (Saltzman et al., 1999; Breiding et al., 2015). As the official definition suggests, most research has focused on the effects of IPV on women, also, child exposure to IPV is often treated different from child maltreatment. Yet, an estimated 10%–20% of children living in the US are annually exposed to IPV (Carlson, 2000). A Canadian incidence study indicated that up to 34% of substantiated investigations into child abuse and neglect were characterized as child exposure to IPV (Trocmé, 2010). Research is exceptionally scarce on the effects of exposure to IPV during the perinatal phase and infancy, even though the assumed harm is significant enough for the WHO to recommend standardized screening for IPV during pregnancy and to call for increased research on prevention for IPV’s adverse effects (World Health Organization, 2011).

Research shows that exposure to adversity over the first 5 years of life can have lasting effects on brain development (Perry, 2002; Fox et al., 2010; Bick and Nelson, 2016). Throughout this sensitive period, interactions with the primary caregivers are a vital learning environment and a primary developmental context. Both positive and negative experiences alike affect the socio-emotional and cognitive development of the child and maturation of associated brain structures (Schore, 2001). Two critical aspects of the interaction with the primary caregivers are essential for brain development: (1) a secure ongoing relationship or attachment between caregiver and child; and (2) a sensitive co-regulation with the caregiver in the presence of a stressor to help the child to develop and increase its capacity for independent emotion regulation. A secure ongoing relationship to a caretaker(s) is fundamental for successful development. Critically, a secure relationship buffers the infant’s hormonal stress response and therefore, protects the developing brain from harmful effects of stress hormones (Gunnar and Donzella, 2001; Tronick, 2017). In contrast, a lack of self-experienced security for the child leads to an increased risk for behavioral problems (Dozier et al., 2001; Belsky and Fearon, 2002) and decreased environmental exploration which compromises the development of cognitive skills associated with school readiness (Moss et al., 1993). Ongoing caregiver relationships that do not provide a sense of safety fail to buffer the hormonal stress responses exposing the infants’ brain to adverse stress effects (Zeanah and Gleason, 2010).

Emotion regulation refers to the child’s ability to modulate and adjust to her levels of arousal (Cole et al., 2004). The frontal lobe, central for the development of emotion regulation, undergoes a period of rapid growth and synaptic excess around 6–18 months of age, making this a critical period for the infant to learn how to respond to emotions (Dawson, 1994; Nelson and Bosquet, 2004). Until self-regulation of emotion is well developed the caregivers are a source of external regulation for the child, hence the caregivers play a vital role in the development of emotion regulatory behaviors. Sensitive responding to the child’s regulatory needs by a caregiver helps the child to regulate stress and to better control stress hormones. This, in turn, helps the child to learn how to regulate herself more effectively. Emotion regulation skills are necessary for the child for learning and focus attention, essential skills to excel in school (Perry, 2001; Cook et al., 2017). By contrast, insensitive caretaking compromises the child’s regulation of arousal and stress. Simply put, a distressed or highly aroused child does not feel safe and is unable to engage with people or objects in the world (Wittling and Schweiger, 1993; Schore, 2001; Cook et al., 2017). Thus, less optimal regulation by a caretaker(s) can become toxic for the child. In sum, these early experiences of sensitive regulation or insensitive maltreatment and dysregulation are critical for the child’s development be it good or ill (Sroufe and Rutter, 1984; Tronick, 2017).

Exposure to IPV can influence both the infant and the caretaker, interfering with the dyadic co-regulation of emotions. IPV can disrupt the caregiver’s ability for optimal caregiving as they may have difficulty regulating their own emotions in the context of violence or are affected by IPV-related psychopathology such as depression and anxiety (Letourneau et al., 2011; Pels et al., 2015). Several studies find that IPV leads to poor mother-infant attachment. Women who experienced IPV during pregnancy or in the first postpartum year had weaker attachments to their infants, perceived their infants as more difficult, and had more doubts about their parenting qualities compared to women not exposed to IPV (Zeitlin et al., 1999; Huth-Bocks et al., 2004; Quinlivan and Evans, 2005). This corroborates research indicating that exposure to IPV is a risk factor for the development and maintenance of secure attachments between mother and child (Sims et al., 1996; Zeanah et al., 1999).

## Exposure to IPV During the Perinatal Period

Increasing evidence indicates that self-reported IPV during pregnancy and the perinatal period is associated with poor health outcomes for the fetus, newborn, and infant up to 1 year postpartum (Cokkinides et al., 1999; Boy and Salihu, 2004; Coker et al., 2004; Rosen et al., 2007; Sarkar, 2008). Exposure to violence increases significant risk factors during the perinatal period, such as a four-times as high risk for antepartum hemorrhage, a condition that can be fatal for the unborn (Janssen et al., 2003; Han and Stewart, 2014). Well established as well are increased risk for low birth weight (Lipsky et al., 2003; Silverman et al., 2006; Rosen et al., 2007), intrauterine growth restriction (Janssen et al., 2003), preterm delivery (Lipsky et al., 2003; Sarkar, 2008), and overall increased fetal morbidity (for review see Boy and Salihu, 2004; Donovan et al., 2016).

Furthermore, maternal high-stress levels during pregnancy, for example, due to exposure to IPV can affect the fetus and its neurohormonal chemistry. The womb is a shared environment with the mother and experiences that affect her can, in turn, affect the developing fetus. For example, the placenta produces an enzyme (11beta-hydroxysteroid dehydrogenase type 2) that breaks down cortisol to an inactive form, protecting the developing fetal brain from its harmful effects. During pregnancy exposure to high-stress contexts increase maternal cortisol along with a downregulation of the enzyme can result in more cortisol reaching the fetus. This exposure can lead to changes in behavioral development (O’Donnell et al., 2009; Davis and Sandman, 2010; Conradt et al., 2013; Ramborger et al., 2018), a larger infant cortisol response, a slower rate of recovery after experiencing a stressor (Davis et al., 2011), as well as make the infant more susceptible to stress later in life (Davis and Sandman, 2010). Conradt found that high stress during pregnancy leads to epigenetic changes in both the mother and the infant and reduced attentional capacities in infants at 4 months of age (Conradt et al., 2013). While only two studies have looked at the stress exposure of women exposed to IPV during pregnancy, both found a significant increase in self-reported stress levels (Chambliss, 2008) and higher levels of the stress hormone cortisol (Han and Stewart, 2014) related to IPV during pregnancy.

## Exposure to IPV During Infancy and Early Childhood

IPV has a high incidence (70%–80%) to occur during the first year postpartum when at least one incident of IPV during pregnancy was reported (Martin et al., 2001; Charles and Perreira, 2007).

### Symptoms of Trauma and Psychopathology

In the absence of language, trauma is difficult to diagnose in young infants. Nonetheless, symptoms reported in infants exposed to IPV are consistent with the definition of trauma in the Zero to Three (Organization) and DC: 0-3R Revision Task Force (2005), which provides diagnostic classification criteria for mental health disorders in infancy and early childhood. Descriptions of infants exposed to IPV include eating problems, sleep disturbances, and mood disturbances (Layzer et al., 1986). Clinical studies find poor sleeping habits, poorer general health, higher irritability, and increased screaming and crying (Alessi and Hearn, 2007). A study looking at multiple forms of traumata in infants, including IPV, found that trauma due to witnessing a threat to a caregiver was related to the most severe symptoms and increased hyperarousal and fear (Scheeringa and Zeanah, 1995; Zeanah and Gleason, 2010, 2015). Moreover, the number of trauma symptoms shows an association with the number of IPV episodes witnessed (Bogat et al., 2006), indicating that an accumulation of trauma symptoms with the accumulation of IPV incidents witnessed by the infant. Next to symptoms of increased arousal, fear, and aggression, interference with development was the most frequently reported symptom of trauma in infants who witnessed severe forms of IPV. For example, the temporary loss of an already acquired developmental skill, such as toilet training or even language. An exceptional study that observed 1-year-old infants in an experimentally simulated situation of adult conflict found that children who previously were exposed to IPV at home as infants showed increased behavioral distress compared to children who had no previous exposure. The finding is indicative of an increased sensitivity to stress as a result of IPV in the first year of life (DeJonghe et al., 2005). Next to a much-needed increase in clinical assessment and longitudinal monitoring of IPV, experimental studies of simulated IPV combined with neurologic and neurohormonal measures on infants and children would greatly advance our understanding how IPV influences the development of regulatory skills and sensitivity to stress.

“Violence becomes traumatic when the victim does not have the ability to consent or dissent, which, in turn, is linked with the universal experience of helplessness and hopelessness engendered by victimization” (Sluzki, 1993, p 179). Exposure to violence, such as IPV, has been recognized as a stressor with a magnitude to produce long-term consequences, including PTSD symptoms in children. Preschool children exposed to IPV show more behavior problems (Hughes, 1988) and significantly lower self-esteem than do older, school-aged children exposed to IPV (Elbow, 1982). Experiencing abusive violence in the home interferes with the child’s developing sense of security and belief in a safe, just world and exceeds the child’s capacity for self-regulation. Evidence shows that exposure to IPV increases the child’s attention towards threatening stimuli, a behavioral pattern that is known to increase the risk to develop internalizing problems, including social and general anxiety, social withdrawal and depression (Kiel and Buss, 2011; Luebbe et al., 2011; Miller, 2015). Externalizing and behavioral problems in children exposed to IPV are also reported to be elevated compared to unexposed (Graham-Bermann and Perkins, 2010). Thus, the age of first exposure to violence along with the cumulative amount of violence witnessed both have a significant effect and increase the risk for the development of externalizing behavior (Graham-Bermann and Perkins, 2010). There is a significant overlap in psychosocial problems of children who either witnessed IPV or were physically abused themselves. A meta-analysis reported that both groups showed significantly more adverse psychological outcomes compared to children who were neither exposed to IPV nor physically abused themselves at home (Kitzmann et al., 2003). In sum, it appears that any violence, including exposure to IPV at home can have detrimental effects on children’s mental health.

### Cognitive Development

Witnessing IPV does not only affect socio-emotional development, several studies have found an impact on a child’s IQ and cognitive functions, such as memory (Jouriles et al., 2008; Graham-Bermann et al., 2010). A study on 1,116 twins found that childhood exposure to IPV was related to a decreased IQ compared to unexposed children, and the severity and number of violent episodes exposed to at home were associated with a greater decrease in IQ. Another study found that children who witnessed IPV on average had an 8-point lower IQ than unexposed children, even when controlling for possible confounding variables suggesting an interplay between trauma-related distress and cognitive skills in children who witnessed IPV at home (Delaney-Black et al., 2002; Koenen et al., 2003). As with emotional development, a longitudinal study found that severe compromising cognitive effects are cumulative and that repeated and increased exposure to IPV was predictive of school engagement (Schnurr and Lohman, 2013).

## Impact of IPV on Brain Development

Adverse childhood experiences, including exposure to IPV, have measurable effects on multiple areas of the brain. Even though there is no study looking directly at the effect of exposure to IPV on the brain during infancy, we can look at retrospective studies of different brain structures maturing during infancy and early childhood. Exposure to adverse experiences, including IPV, affects the development of the Hypothalamus-Pituitary-Adrenal (HPA) axis and brain structures related to witnessing itself (auditory and visual cortex).

### The HPA Axis

Adversity has been reported to affect the development of the HPA Axis. The HPA axis is a critical stress response system, enabling appropriate responding to stressors and the return of the body to homeostasis. While this stress response is essential and helpful to adapt to everyday stressors appropriately, a chronic activation due to chronic exposure to stress can predispose to psychological, immune and metabolic alterations, and associated detrimental effects due to exposure to excess glucocorticoids. In infancy and childhood, the HPA axis and cortisol reactivity are still maturing (Gunnar and Donzella, 2001), making the system vulnerable to adverse experiences (Tarullo and Gunnar, 2006). More important, changes due to high-stress exposure during this critical time of maturation may not only be long-lasting but also be harder to treat as a normal functioning may never have been established.

During infancy and early childhood, the production of the stress-hormone cortisol appears to be buffered and insensitive to a number of stressors. In both, humans and rodents, maternal caregiving has been identified as a primary factor in the infants HPA hyporesponsivity (Lupien et al., 2009). This buffering effect through the caregiver likely protects the infant brain from the harmful effects of high levels of cortisol and is therefore even more critical in high-stress environments such as ones in which there is chronic exposure to IPV. In humans, sensitive caregiving and co-regulation have been shown to contribute to lower levels of cortisol, while lower quality of care or insecure attachment, as often reported for children with exposure to IPV, are often associated with elevated levels of the stress-hormone (Spangler and Grossmann, 1993; Nachmias et al., 1996; Dettling et al., 2000; Ahnert et al., 2004; Müller et al., 2015). One study reported that salivary cortisol levels in 1-year-old infants are negatively correlated with electroencephalogram (EEG) potentials, indicating that brain activity is directly affected by elevated levels of cortisol (Gunnar and Nelson, 1994). Chronic high levels of cortisol lead to cell death, especially in those brain structures with a high density of glucocorticoid receptors (Virgin et al., 1991). For example, cell death has been found in humans taking high-dose cortisol medication (e.g., for asthma). Adults and children showed decreased verbal memory, and a decline in explicit memory, both cognitive functions that are related to the hippocampus (Bender et al., 1991; Newcomer et al., 1994), and the observed effects were dose-dependent. That a medical form of cortisol can have such a severe impact on cognitive performance strongly suggests that stress-linked cortisol concentrations due to chronic exposure to IPV or any high-stress environment can have harmful consequences for the developing brain of the infant.

### Auditory and Visual Cortex

Witnessing even just verbal abuse between caregivers as part of IPV, without physical violence, can have observable impacts on the developing brain. Magnetic resonance imaging (MRI) scans show differences in gray matter density in the arcuate fasciculus in the left superior temporal gyrus, an area involved in language processing, with a reduction in young adults who reported witnessing parental verbal abuse starting at the age of 3–13 years. In a similar sample, diffusion tensor imaging (DTI) scans found a significant reduction of white matter volume in temporal gyrus associated with exposure to verbal abuse. Critically, these reductions showed a significant correlation to verbal IQ and language comprehension (Choi et al., 2009; Tomoda et al., 2011).

The visual cortex processes emotional stimulation and information. Strikingly, repeated visual exposure to IPV was related to reduced volume in the visual cortex and diminished connections between visual cortex and limbic system. Most important, the observed reductions in brain volume and intra-neuronal connections were directly associated to the chronicity of exposure before the age of 12 (Choi et al., 2012; Tomoda et al., 2012). These findings indicate that early exposure to IPV, such as witnessing verbal abuse between caregivers could have affected the integrity of specific brain structures.

## Summary and Future Directions

The goal of this mini-review was to examine the evidence of the impact of exposure to IPV during the perinatal phase through early childhood. The definition of IPV is adult focused and is subsumed by other (poorly defined) terms (e.g., neglect, maltreatment). In contrast to neglect, witnessing IPV occurs when a caregiver is present and is distinct from violent maltreatment, as when a child is exposed to IPV the witnessed violence is not directed against the child. These kinds of maltreatment are likely to affect the child differently in physical terms and psychological terms. For example, how the child cognitively processes each of those experiences may radically differ. Moreover, IPV and other forms of mistreatment almost always co-occur, making it problematic to identify singular effects specific to IPV. These problems make it difficult to evaluate many of the studies for the effects of IPV separate from other forms of mistreatment. Of course, in the real world, exposure to IPV is in actuality an assemblage of developmentally disruptive actions which will most often have multiple physical and psychological effects on the child.

One hypothesis on how IPV affects development can be derived from a developmental framework: witnessing IPV depletes resources that normally would be—should be—utilized for growth and development (Hobfoll, 1989; Tronick, 2017). It is well established that secure and sensitive caregiving is fundamental for the favorable development of brain structures associated with regulatory capacities. Such caretaking is, essential for the child to develop behavioral self-regulation. The occurrence of IPV disrupts this favorable caregiver-child interaction while it is happening, but critically not only while it is occurring. Moreover, adults on both sides who are involved in IPV likely have much more pervasive problems, perhaps due to their own exposure to IPV. Critically for the child, their experience of IPV affects their everyday caretaking (Letourneau et al., 2011; Pels et al., 2015). The effect of the caretaker’s state after IPV means the child has not only witnessed IPV but that the caretakers likely lack the resources for sensitive and consistent caregiving. Thus, they may be unable to help the child regulate or provide a sense of safety when the child needs it most, during or after witnessing an episode of violence.

Disruption in caregiving is prevalent, and families dealing with IPV most likely are affected by co-occurring conditions such as parental mental health problems associated with the abuse, such as depression and anxiety or inconsistency in caregiving due to IPV related circumstances. More research and a more thorough distinction between different forms of adversity are needed to understand how exposure to IPV distorts the functioning and development of regulation-associated brain systems. DeJonghe et al. (2005) make an essential step towards a more profound understanding how sensitivity to stress as a result of IPV develops in the first year of life as theirs is the only experimental study in humans looking at behavioral effects of acute exposure to violence in infants that either have or have not been exposed to IPV. An extension of experimental research could assist in gaining a deeper understanding of how IPV disrupts caregiving, successful co-regulation with the caregiver to cope with an acute or chronic stressor, and the development of self-regulation in infancy. Examining the effects of IPV on physiological and endocrine markers of stress, as well as imaging methods documenting the effects of exposure to violence on infants in experimental as well as descriptive studies will help to separate primary and collateral causes that disrupt the child’s functioning and healthy development. And while much needs to be learned about IPV, the available evidence makes it clear that policies for prevention of witnessing IPV and experiencing other forms of maltreatment are critically needed for the insuring well-being of our infants and young children.

## Author Contributions

IM contributed to this manuscript by selecting and summarizing relevant studies and writing multiple sections. IM and ET contributed to the writing and editing of the review article.

## Conflict of Interest Statement

The authors declare that the research was conducted in the absence of any commercial or financial relationships that could be construed as a potential conflict of interest.
